# Brain-Derived Extracellular Vesicle Subpopulations: from Bulk Measurements to Single-Entity Assays

**DOI:** 10.1021/jacsau.6c00300

**Published:** 2026-05-10

**Authors:** Shiyao Bai, Leyi Chen, Wenjing Zhang, Mingyuan Li, Han Liu, Xiaowen Huang, Lianqun Zhou, Weini Xin, Cheng Jiang, Chen-zhong Li

**Affiliations:** † Department of Biomedical Engineering, School of Medicine, 407605 The Chinese University of Hong Kong, Shenzhen 518172, China; ‡ Guangdong Basic Research Center of Excellence for Aggregate Science, School of Science and Engineering, The Chinese University of Hong Kong, Shenzhen 518172, China; § Institute of Translational Medicine, Shanghai University, Shanghai 200444, China; ∥ Institute of Brain Science and Brain-Inspired Research, Shandong First Medical University and Shandong Academy of Medical Sciences, Jinan, Shandong 250000, China; ⊥ State Key Laboratory of Biomedical Imaging Science and Systems, Suzhou 215163, China; # Suzhou Institute of Biomedical Engineering and Technology, Chinese Academy of Sciences, Suzhou 215163, China; ¶ Hospital of Stomatology, 66477 Shantou University Medical College, Shantou 515041, China

**Keywords:** brain derived extracellular vesicles (BDEVs), neurodegenerative diseases (NDs), early diagnosis, biomarkers, EV detection

## Abstract

Neurodegenerative diseases (NDs), such as Alzheimer’s disease (AD) and Parkinson’s disease (PD), pose a significant global health challenge, currently affecting over 40 million individuals and placing a heavy burden on healthcare systems. Existing diagnostic methods, such as neuroimaging and cognitive assessments, often lack sufficient sensitivity and specificity, especially in the early stages of disease. This underscores the need for novel biomarkers. Extracellular vesicles (EVs), particularly those derived from the brain, i.e., brain-derived extracellular vesicles (BDEVs), hold great potential as noninvasive diagnostic tools due to their ability to reflect the physiological and pathological states of their cells of origin. However, isolation and detection of such EV subpopulations from accessible body fluids such as blood remain a technical challenge due to their low abundance and overlapping physical properties compared to other EVs. This review discusses rationally designed isolation and detection technologies for EVs from major brain cell subpopulations and their integration with emerging fields like AI and big data analysis. We specifically contrast traditional ensemble-averaged bulk measurements with emerging single-entity assays, highlighting how the latter bypass biological noise to resolve rare BDEV subpopulations. It highlights the potential of these EV subpopulations as biomarkers, addresses EV isolation challenges and proposes standardized methodologies, and emphasizes the need for comprehensive profiling of EV markers at single-entity level, and point-of-care testing (POCT) development.

## Graphical abstract

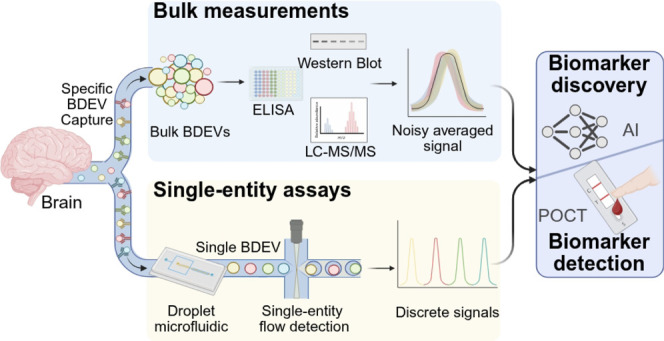

## 1 Introduction

The escalating global prevalence of neurodegenerative diseases (NDs), primarily Alzheimer’s disease (AD) and Parkinson’s disease (PD), has created a critical demand for early and precise diagnostic strategies.^1–5^ These conditions are molecularly rooted in the progressive accumulation of pathological protein aggregates, such as β-amyloid (Aβ) and α-synuclein (α-syn), which ultimately trigger irreversible neuronal dysfunction.^6,7^ Standard clinical modalities, including neuroimaging and cognitive profiling, provide essential diagnostic insights but fall short of the molecular sensitivity needed for early-stage detection.^8,9^ In contrast, while cerebrospinal fluid analysis offers exceptional diagnostic reliability, its inherent invasiveness restricts its use for routine population screening or longitudinal monitoring.^10^

Beyond cerebrospinal fluid, other systemic liquid biopsy candidates such as circulating tumor cells (CTCs) and cell-free DNA (cfDNA) have been explored for disease monitoring.^11^ However, they face significant limitations in the context of neurodegeneration. While cfDNA and CTCs typically represent terminal cellular events such as apoptosis, genomic instability, or physical shedding, brain-derived extracellular vesicles (BDEVs) are actively secreted by living cells.^12,13^ This active biogenesis ensures that BDEVs encapsulate a diverse and protected cargo of proteins and functional RNAs. These molecules mirror the real-time physiological status of their parental CNS cells rather than merely reflecting metabolic remnants.^14^ Most importantly, the unique capacity of BDEVs to traverse the blood–brain barrier (BBB) and enter systemic circulation provides a noninvasive “window” into the cerebral molecular landscape. This intrinsic transport mechanism represents a diagnostic capability that CTCs and cfDNA generally lack in the context of brain-specific pathologies.

Therefore, the identification of extracellular vesicles (EVs) as biological nanocarriers offers a promising resolution to these diagnostic constraints.^15,16^ These lipid-bilayer nanoparticles are secreted by virtually all brain cell types,^17,18^ serving as molecular messengers that enable high-fidelity sampling of the CNS through a routine blood draw.^6,7,18,19^ However, the practical implementation of BDEV-based diagnostics is hindered by their extreme scarcity within the peripheral environment.^13^ In human plasma, BDEVs are overwhelmed by a vast background of vesicles originating from platelets and endothelial cells, a situation that creates a formidable signal-to-noise challenge. Traditional separation techniques based on density or size are frequently inadequate because BDEVs share overlapping physical properties with the vast majority of non-brain EVs.^15,20^ This biochemical overlap necessitates the development of sophisticated chemical strategies for targeted enrichment and ultrasensitive detection, shifting the analytical focus from ensemble-averaged bulk measurements toward high-resolution assays capable of resolving single vesicles.^21^

In this review, we provide a systematic evaluation of the technological landscape for probing brain EV subpopulations, structured around the exacting analytical requirements for clinical translation. As illustrated in Figure 1, we establish a comprehensive analytical framework tracking the translational trajectory of BDEVs. Briefly, diverse brain cells secrete specific BDEV subpopulations carrying distinct surface antigens into the peripheral blood. Based on these criteria, we first examine the chemical principles underlying current separation and enrichment strategies for isolating cell-specific vesicles, followed by an assessment of the characterization and origin-tracing methodologies used to authenticate their neural identity. Central to this discussion is the analytical evolution from ensemble-averaged bulk measurements toward emerging single-entity assays (e.g., electrical, optical, and electrochemical platforms), which offer the resolution necessary to decode the inherent heterogeneity of individual vesicles. Furthermore, by integrating the high-dimensional data generated from these sensing platforms with artificial intelligence (AI) algorithms, we highlight how this framework accelerates the robust discovery of novel biomarkers. By synthesizing these technological advancements and addressing the persistent challenges in molecular recognition and signal transduction, this review aims to provide a strategic roadmap for transitioning BDEV-based diagnostics from the laboratory into routine clinical practice and point-of-care testing (POCT).

**Figure 1:**
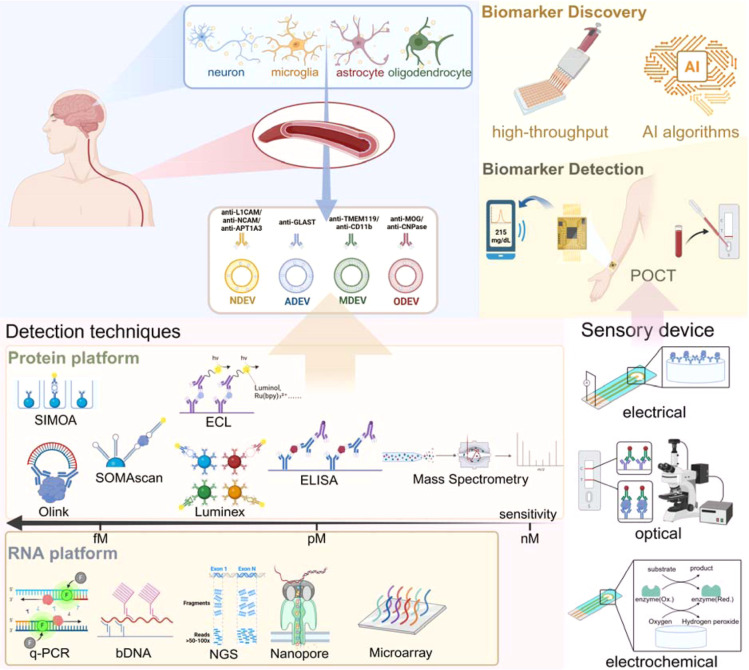
Overview of biomarker discovery and detection in neurodegenerative diseases.

## 2 Standardized Performance Metrics for Benchmarking BDEV Technologies

A major obstacle in profiling brain extracellular vesicle subpopulations is that evaluation end points and sample conditions are not standardized across technologies. The absence of harmonized standards hinders direct benchmarking between technologies, leaving the true validity of reported sensitivity and specificity in question.^22^ To enable a more consistent assessment from bulk measurements to single-entity assays, this review adopts a unified analytical framework centered on two core questions: (i) whether rare BDEV signals can be detected reliably in complex biofluids, and (ii) whether EV heterogeneity can be resolved into biologically meaningful subpopulations rather than ensemble-averaged readouts. In the following sections, we apply the same set of performance metrics and explicitly consider workflow-dependent sources of bias when evaluating each methodological module.^23^

BDEV subpopulation profiling depends on a core set of performance metrics, primarily the limit of detection (LoD) and analytical resolution. LoD defines the smallest signal that can be told apart from background noise, which is a vital parameter because brain markers are highly diluted in the systemic circulation and require ultrasensitive detection modalities to be identified amidst a vast background of peripheral vesicles.^24^ Analytical resolution marks the move from group-average measurements to detecting single particles. Unlike bulk assays that hide differences with average values, single-particle detection finds the detection frequency and colocalization needed to show that cargo and origin markers are in the same vesicle, rather than being separate impurities.^25^ This shift is necessary to establish the stoichiometric relationship between different surface antigens and internal cargo within a single entity.

Specificity further needs a multilevel approach across physical, molecular, and biological stages. This framework must first tell vesicles apart from other particles such as lipoproteins or exomeres that share similar size and density profiles.^26–28^ The second stage involves reducing cross-reactivity for accurate marker detection, followed by the use of multiple markers to verify a brain cell origin. This layered analysis is critical to account for the presence of nonvesicular protein isoforms, which can lead to the significant overestimation of brain-derived concentrations in human plasma.^29^ By implementing these verification layers, researchers can ensure that results truly reflect brain pathology instead of the massive biological noise in the blood.

Even with good performance metrics, the reliability of BDEV profiling can be biased by factors in both preanalytical and analytical stages.^30^ Preanalytical steps, such as collection methods, processing time, and storage, can damage EV integrity or add background noise.^31^ Moreover, the trade-off between recovery and purity during isolation can change the vesicle population before it ever reaches the sensor. During measurement, further errors may come from epitope accessibility and nonspecific binding. Single-particle platforms might also favor larger or brighter vesicles, leading to the loss of rare signals in the final data. Therefore, using the right controls, such as assay blanks and batch-to-batch calibration, is essential to ensure that results reflect real biological differences instead of experimental artifacts.

## 3 Separation of BDEV Subpopulations

As critical carriers of pathological signatures, the research and application of BDEVs rely on the efficient and specific separation of target vesicles from complex biological samples such as blood, cerebrospinal fluid (CSF), and brain tissues. The quality of the isolated EV samples directly determines their accuracy in downstream analyses and diagnostic applications. In the context of our established analytical framework, separation serves as the first gatekeeper of specificity, particularly at the physical stage where vesicles must be distinguished from nonvesicular nanoparticles. However, the choice of a separation strategy involves a critical trade-off between recovery and purity, which directly dictates the downstream LoD and the validity of subpopulation-specific signals. While the field has developed a diverse toolkit for EV isolation, current methodologies must be critically evaluated based on their ability to meet the benchmarking standards required for high-fidelity BDEV profiling.

### 3.1 Physical Property-Based Separation

Traditional isolation methods primarily exploit differences in size, sedimentation velocity, and buoyant density between EVs and other components. Ultracentrifugation (UC), an isolation method based on sedimentation coefficients, has been widely employed in the studies such as those by Morgan C. Pait et al., who isolated EVs from the hippocampal interstitial fluid (ISF) of live mice to explore the Alzheimer’s disease-related biomarkers contained within them.^32^ However, UC lacks the resolution to differentiate BDEVs from proteins or other vesicular components with similar sedimentation coefficients, which is particularly problematic in human plasma where rare brain-derived signals are masked by a massive background of peripheral particles. Furthermore, the ultrahigh centrifugal forces up to 100,000*g* can cause structural damage to EVs, introducing preanalytical bias that compromises the integrity of the vesicles before they reach the detection stage.

To mitigate these issues, density gradient centrifugation (DGC) utilizes media like iodixanol or sucrose to achieve higher resolution under gentler conditions. Compared with UC, DGC removes protein contaminants more effectively and mitigates EV loss caused by mechanical compression due to the cushioning effect of the gradient medium. The Stephanie L. Fowler team employed DGC to isolate EVs derived from the brain tissue of patients with Alzheimer’s disease and further investigated the molecular species and structural characteristics of assembled tau associated with these EVs.^33^ Despite these advantages, DGC still struggles to completely exclude specific lipoprotein subclasses and exomeres that share similar density profiles with small EVs.^27,28^

Other physical strategies include size-exclusion chromatography (SEC), which Marie Oosterlynck et al. combined with UC to isolate human brain-derived EVs (Figure 2A).^34^ However, this technique is still limited by coisolating similarly sized lipoproteins. Polymer-based precipitation represents another common strategy using polymers like polyethylene glycol (PEG) to reduce EV solubility. Similarly, polymer precipitation is operationally simple but frequently yields substantial impurities; for instance, Jente Schmeetz et al. demonstrated that the use of precipitation for isolating EVs from simple samples like tears does not preclude the risk of coisolating macromolecular aggregates^35^ (Figure 2B). Within our benchmarking framework, the reliance on size alone in these methods is often insufficient to capture rare signals in systemic circulation where biological noise is significantly higher.

**Figure 2:**
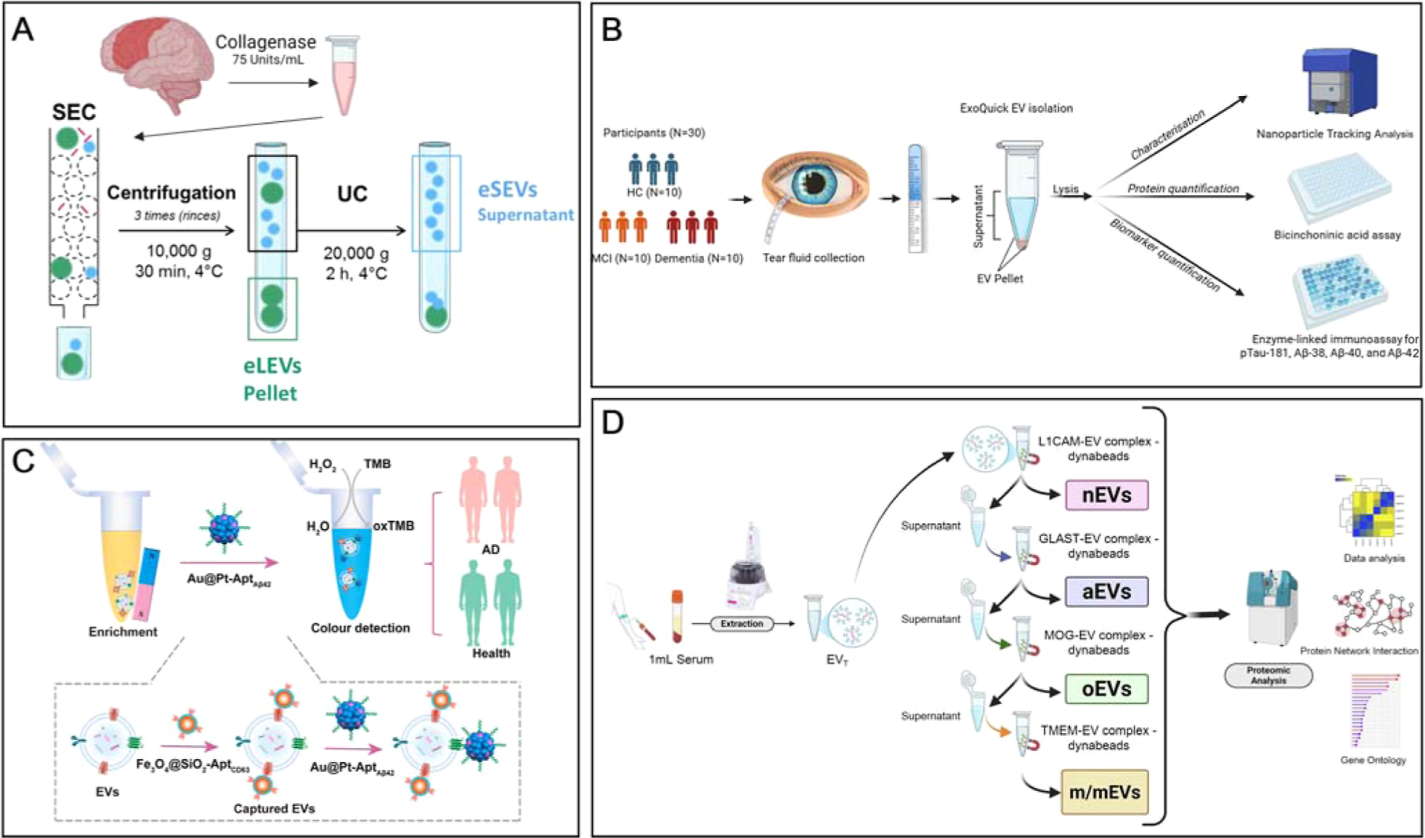
(A) Flow process for isolating EVs from brain tissue using SEC and UC. Adapted from ref 34 Available under a CC-BY 4.0 license. Copyright 2025 Springer Nature. (B) Flowchart for isolating extracellular vesicles from tear samples using the ExoQuick reagent kit (PEG precipitation method). Adapted from ref 35 Available under a CC-BY 4.0 license. Copyright 2025 Elsevier. (C) Flowchart for the preparation of aptamer-modified immunomagnetic beads and the capture of EVs. Adapted with permission from ref 40 Copyright 2026 Elsevier. (D) Flow process and principal diagram of capturing 4 subtypes of BDEVs using polymer precipitation and immunoaffinity magnetic beads. Adapted with permission from ref 36 Copyright 2025 Springer Nature.

### 3.2 Biochemical Affinity-Based Enrichment: Targeted Subpopulation Capture

To resolve BDEV subpopulations from complex mixtures, the field has shifted toward targeted enrichment using specific membrane protein markers. As comprehensively compared in Figure 2, while traditional physical isolation methods (e.g., ultracentrifugation and size-exclusion chromatography; Figure 2A) are inherently limited by their lack of cell-type specificity and often result in heterogeneous bulk mixtures, emerging affinity-based and magnetic enrichment platforms (Figure 2C,D) offer a paradigm shift. These innovative strategies enable the direct, rapid, and highly specific capture of BDEV subpopulations from complex clinical biofluids, significantly improving the purity and yield required for downstream diagnostics. Immunoaffinity approaches, typically leveraging antigen–antibody interactions, allow for the selective capture of target vesicles from total EV populations. For example, the Laura Camacho-Meño team used magnetic labeling with L1CAM, GLAST, MOG, and TMEM-119 antibodies to isolate extracellular vesicles secreted by neurons, astrocytes, oligodendrocytes, and microglia, respectively (Figure 2D).^36^ The selection of these surface markers is fundamental to achieving high-fidelity enrichment across different CNS lineages. These lineage-specific markers provide essential molecular handles for probing CNS health through systemic biofluids. However, as emphasized in our benchmarking framework, the use of a single marker faces a specificity crisis. The discovery that soluble L1CAM isoforms can lead to the overestimation of BDEV concentrations in human plasma suggests that targeted isolation must move toward more rigorous biological verification.^29^ To overcome this, the field is transitioning from single-marker capture toward “antibody cocktail” strategies and multidimensional verification. By combining lineage-specific markers with generic tetraspanins (CD9/CD63/CD81) and employing single-entity imaging to verify marker colocalization, researchers can effectively extract genuine brain-pathology fingerprints from the background noise of systemic circulation. In addition, several studies have focused on developing novel biomarkers with enhanced specificity for BDEVs, such as ATP1A3,^37^ a marker specific to neuron-derived extracellular vesicles (NDEVs). The ability of these biomarkers to enable the isolation of NDEVs has subsequently been validated and applied in recent studies.^38^

In response to these challenges, next-generation ligands such as nanobodies and aptamers are increasingly being applied to BDEV isolation. The Marija Tursunović team identified three nanobodies (NA8, ND101, and ND102) capable of specifically capturing extracellular vesicles, demonstrating excellent stability and the ability to access epitopes that are inaccessible to conventional antibodies.^39^ Similarly, aptamer-based technologies, such as the CD63-aptamer modified magnetic particles utilized by Junli Zhang et al., capture EVs through specific affinity interactions while aiming to reduce the nonspecific binding that often compromises subpopulation profiling (Figure 2C).^40^ At the current stage, the application of nanobodies and aptamers in EV isolation has predominantly targeted vesicle-specific membrane proteins, such as CD63. However, both technologies enable the generation of high-affinity and high-specificity nanobodies or aptamers against selected targets through library construction and screening strategies.^41,42^ Therefore, they hold considerable potential for application in the capture and isolation of BDEVs. Furthermore, molecular ligands originally designed for therapeutic purposes or single-molecule protein detection (e.g., Simoa) are being explored for EV separation due to their high specificity and potential for automated synthesis.^43^ While these targeted approaches provide a bridge toward single-entity assays, they must be carefully calibrated to avoid favoring only the largest or most fluorescently bright vesicles in a population.

### 3.3 Challenges in EV Isolation and Future Trends

Although the development of isolation technologies is relatively mature, the separation of high-purity BDEV subpopulations still has limitations. Traditional physical methods lack specificity when processing complex samples such as serum and plasma and are more suitable for nerve cell culture supernatants, brain tissue homogenates, or CSF. While affinity capture improves specificity and purity, it is constrained by high costs, reliance on high-quality ligands, and the lack of clear biomarkers for some subpopulations. Limited specificity can lead to underrepresentation or distortion in subpopulation biomarker profiling and disease detection results. Meanwhile, purity variations introduced by different isolation strategies may mask or interfere with low-abundance candidate biomarker signals during downstream protein or nucleic acid analyses. In addition, many existing methods leave the EVs bound to a solid phase, and common release steps often damage the vesicles or alter their molecular cargo, which introduces downstream analytical bias and hinders standardization.

Faced with these challenges, BDEV separation technology is moving toward milder, automated, and standardized approaches to meet the needs of high-throughput analysis. To overcome the structural damage caused by traditional affinity elution methods such as extreme pH changes, emerging mild release strategies have gained attention. For example, based on the design of stimulus-responsive chemical cleavage^44^ or photoresponsive systems,^38,45^ captured EVs can be released efficiently under mild triggering conditions. These methods significantly improve the structural integrity and biological activity of released EVs, providing a reliable foundation for downstream biomarker analysis. Other strategies employ functionalized liposomes to capture target EV subpopulations by membrane fusion.^46^ Because nonbiological materials are used, the isolated EVs can be directly subjected to downstream biomarker profiling and analytical assays, eliminating additional release steps. In terms of automation, microfluidic chip-based technologies incorporating advanced driving mechanisms such as dielectrophoresis^47^ and alternating magnetic fields^48^ demonstrate substantial potential. By integrating complex separation steps onto miniaturized chips, these platforms reduce manual errors and improve reproducibility, addressing the preanalytical variables that compromise cross-study comparability. These trends move the field away from ensemble-averaged bulk isolation toward the analytical resolution needed for the single-entity assays that will be explored in the following sections.

## 4 Evaluation of Brain EV Subpopulations

Robust evaluation of brain EV subpopulations requires a two-layer quality-control logic designed to bridge the gap between initial isolation and downstream biomarker discovery. First, regardless of the intended detection modality, the isolated material must be verified as EV-enriched preparations rather than mixtures dominated by nonvesicular nanoparticles and soluble contaminants. This stage of generic evaluation addresses the physical and molecular specificity requirements of our analytical framework, ensuring that the isolated entities possess the characteristic membrane structure and size distribution of EVs.^49,50^ Second, because brain-derived EVs in peripheral biofluids are typically present at low abundance and are difficult to enrich by physical-property-based methods, additional evidence is required to support the cell-type origin and purity of the targeted subpopulation.

Indeed, conventional isolation approaches such as ultracentrifugation,^51^ size-exclusion chromatography,^52^ and polymer-based precipitation^53^ rely primarily on size and density. As discussed in the previous sections, these physical metrics are often insufficient for subpopulation enrichment due to the significant overlap in biophysical properties between EVs from different cellular origins. While immunoaffinity capture is frequently employed to resolve this heterogeneity, its performance remains critically dependent on the specificity of the antibody or aptamer used.^54^ In this context, evaluation must move beyond simple marker presence to assess the stoichiometric congruence between surface antigens and internal cargo. This multilevel verification is essential to mitigate the risk of analytical bias and to ensure that the detected signals represent the genuine pathological state of the central nervous system rather than coisolated artifacts from systemic circulation.

### 4.1 Generic Evaluation

As emphasized by the ISEV minimal-information frameworks (MISEV2018 and the updated MISEV2023),^55,56^ generic characterization should combine orthogonal readouts that collectively establish (i) vesicular morphology, (ii) particle size/concentration, and (iii) EV-associated protein signatures, while also documenting preanalytics and separation details to support reproducibility and cross-study comparability.

At the structural level, transmission electron microscopy (TEM) or cryo-electron microscopy provides direct evidence of vesicle-like morphology and can help identify gross contaminants (e.g., amorphous protein aggregates).^57,58^ Although morphology alone is not sufficient to define EV identity, EM remains a widely adopted “first-pass” verification tool when interpreted together with particle-counting and molecular marker data.

Quantitatively, nanoparticle tracking analysis (NTA) is commonly used to report particle size distributions and concentration, offering a practical readout for yield comparison across isolation methods and for batch-to-batch QC.^59^ In brain-EV subpopulation studies, NTA can be further extended to fluorescence-enabled formats to evaluate labeled/immunocaptured fractions, consistent with reports that combined immunocapture and fluorescent nanoparticle tracking has been used in subpopulation-oriented analyses.

At the molecular level, Western blot (WB) (or equivalently validated immunoassays) is typically used to confirm the presence of EV-enriched protein markers and to assess depletion of nonvesicular components. Notably, the updated MISEV2023 ^56^ mandates a more rigorous authentication approach than the 2018 version^55^ by explicitly addressing EV heterogeneity and coisolated particles. For rare targets like BDEVs in complex peripheral blood, adhering to these stringent requirements is now an absolute prerequisite. Researchers must demonstrate the copresence of luminal (e.g., TSG101/ALIX) and transmembrane (e.g., CD9/CD63/CD81) markers while explicitly depleting nonvesicular coisolates (a term updated from “contaminants”). Furthermore, MISEV2023 strongly encourages the transition toward single-EV characterization, perfectly aligning with the analytical evolution advocated in this review. This updated standard ensures the true vesicular nature and authentic neural origin of BDEVs prior to downstream analysis.

In practice, commonly used EV-enriched markers include tetraspanins (CD9/CD63/CD81) and endosome-associated proteins (e.g., TSG101/ALIX), whereas “negative/depleted” markers are selected to reflect likely contaminants for the sample type (e.g., ER/Golgi-associated proteins for cellular debris; in plasma/serum, attention to abundant protein and lipoprotein coisolates is also important).

Finally, for brain-EV subpopulation studies specifically, generic characterization is best interpreted together with the upstream enrichment strategy. Many studies enrich neuron- or oligodendrocyte-associated fractions using cell-type markers (e.g., anti-L1CAM for NDEVs and anti-MOG for oligodendrocyte-derived extracellular vesicles [ODEVs]), after which generic QC readouts (EM/particle metrics/marker panels) provide critical context for whether observed biomarker differences likely reflect genuine subpopulation biology versus variability in isolation efficiency and coisolate burden.

### 4.2 Biological Origination Evaluation

The fundamental challenge in the clinical translation of BDEVs lies in the rigorous authentication of their biological origin. Traditionally, this evaluation has relied on the assumption of molecular congruence between the vesicle membrane and its parent CNS cells, employing surface markers such as L1CAM and NCAM for neurons, or GLAST and AQP4 for astrocytes.^60^ However, the reliability of single-marker immunoaffinity enrichment has recently come under intense scrutiny. As reported by Norman et al., L1CAM—the long-standing “gold standard” for NDEVs—exists significantly in a soluble, nonvesicular form in human plasma, which leads to substantial cross-contamination and the overestimation of BDEV concentrations.^61^ This analytical challenge is further compounded by the intrinsic heterogeneity of EV biogenesis. Kowal et al. demonstrated through proteomic comparison that EVs released by the same cell type comprise distinct subpopulations, such as endosome-derived exosomes and plasma-membrane-budded microvesicles, which harbor divergent protein signatures. For instance, while tetraspanins like CD9, CD63, and CD81 are common markers, their relative abundance varies significantly across EV subtypes, suggesting that a single surface antigen may only capture a specific fraction of the total BDEV pool.^62^

Furthermore, the physiological baseline of circulating EVs is highly dynamic. Holcar et al. emphasized that EV composition in healthy humans is influenced by sex, age, and circadian rhythms, contributing to a “biological noise” that must be characterized before pathological BDEV subpopulations can be accurately traced.^63^ Consequently, the field is transitioning toward systematic, multidimensional methodologies that integrate physical, chemical, and computational evidence. Liu et al. noted in a recent systematic review that high-fidelity subpopulation analysis now necessitates the integration of high-efficiency separation microfluidics with ultrasensitive detection modalities to resolve these heterogeneous signatures.^64^ As highlighted in the workflows of Figure 3, the core technological innovation here lies in the transition from relying on single surface markers to employing multiomics cross-validation. By integrating RNA-based digital deconvolution with protein-based dual-layer verification, these advanced verification strategies provide a more rigorous and multidimensional confirmation of the BDEVs’ neural lineage, effectively minimizing false-positive signals from peripheral EV interference.

**Figure 3:**
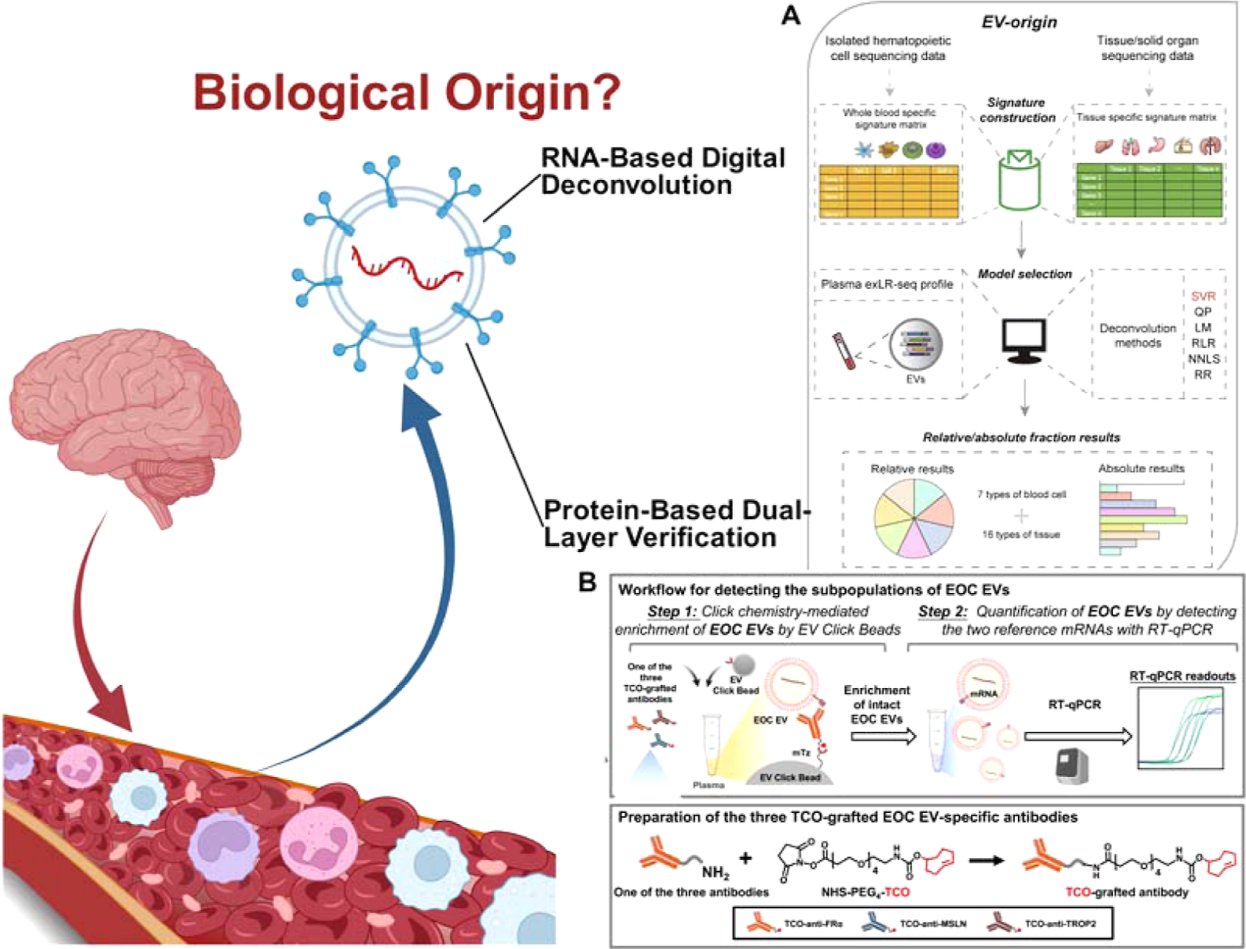
Biological origination evaluation of BDEVs. Rigorous authentication is essential to resolve BDEV heterogeneity and distinguish CNS-derived signals from systemic background noise. (A) RNA-based digital deconvolution: A computational approach that utilizes exLR-seq profiles as a “genetic mirror” to mathematically resolve the tissue-cellular origins of circulating EVs. Adapted from ref 68 Available under a CC-BY 4.0 license. Copyright 2020 Elsevier. (B) Protein-based dual-layer verification: An experimental cascade employing a “surface-capture/internal-readout” logic. Captured vesicles are verified by internal lineage-specific transcripts to ensure neural identity and filter out soluble protein contaminants. Adapted from ref 69 Available under a CC-BY 4.0 license. Copyright 2020 Elsevier.

Currently, this systematic authentication is achieved through two primary technological pillars. The first is RNA-based digital deconvolution, exemplified by the EV-origin computational framework.^65^ Given that EVs encapsulate a protected cargo of long RNAs (exLRs) that serve as a “genetic mirror” of their parent cells, optimized exLR-seq can reveal over 10,000 RNA species, including mRNA, circRNA, and lncRNA.^66^ By applying ν -SVR deconvolution algorithms to these profiles, the total EV population in blood can be mathematically resolved into its constituent tissue-cellular sources, allowing for the quantitative tracking of CNS-derived fractions even amidst a dominant background of platelet-derived EVs^67,68^ (Figure 3A).

The second pillar involves protein-based dual verification using platforms such as EV Click Chips.^69^ To overcome the low specificity of single markers, these systems employ an “antibody cocktail” targeting multiple lineage-specific antigens combined with bio-orthogonal click chemistry for synergistic, covalent capture. This is followed by a second layer of authentication where the captured subpopulation is analyzed for tissue-lineage associated transcripts (e.g., MAP2, NEFL) via RT-ddPCR^64,69^ (Figure 3B). This “surface-capture/internal-readout” cascade effectively filters out nonspecific background noise and soluble protein contaminants. By integrating proteomic subtype characteristics, physiological baseline modeling, and dual-layer molecular authentication, these systemic strategies provide the necessary resolution to decode the inherent heterogeneity of BDEVs, paving the way for their use as high-fidelity liquid biopsy tools in neurology.

Through the application of these multidimensional verification strategies, a clearer comparative landscape of BDEV subpopulations emerges. While NDEVs, astrocyte-derived extracellular vesicles (ADEVs), microglia-derived extracellular vesicles (MDEVs), and ODEVs share the common analytical challenge of extreme scarcity amidst a dominant background of peripheral vesicles, their diagnostic utility is defined by distinct molecular profiles. NDEVs and ODEVs, for instance, are the primary vehicles for detecting CNS-derived pathological aggregates like α-syn, whereas ADEVs are typically characterized by structural proteins such as GFAP or GLAST, reflecting neuroinflammatory states through cargo such as C1q. Furthermore, MDEVs provide a unique genetic window through specific protective or pathogenic RNA signatures, such as circZNRF1 or microRNAs. This differentiation underscores that while the authentication methods discussed here are broadly applicable, the successful clinical translation of each subpopulation depends on aligning these lineage-specific biological fingerprints with the high-resolution detection technologies explored in the following sections.

## 5 Analytical Strategies for Detecting Brain-Derived EV Subpopulations

After establishing the cellular origins and biological roles of BDEVs, the focus shifts to the analytical strategies required to detect and quantify these rare messengers in systemic biofluids. Traditionally, this analysis has relied on bulk detection techniques that provide an ensemble-averaged view of molecular signatures.^70–72^ While these methods are essential for mapping general secretion dynamics and cargo transmission,^70,73,74^ they often sacrifice analytical resolution and require large volumes of precious clinical samples.^75,76^ To overcome these barriers, the field is moving toward single-entity and microfluidic platforms that offer higher sensitivity and the ability to resolve discrete vesicle heterogeneity. This section evaluates the current landscape of BDEV detection, benchmarking the discovery power of established bulk technologies against the precision of emerging single-vesicle tools through the lens of sensitivity, specificity, and sample efficiency. A comprehensive benchmarking of these diverse detection platforms, detailing their analytical resolution, sensitivity, and clinical limitations, is summarized in Table 1.

**Table 1:** Performance Comparison of Analytical Platforms for BDEV Detection

technology category	platform	sensitivity (LoD)	analytical resolution	multiplexing capability	cost	clinical applicability	limitation	
Bulk immunoassay	ELISA	ng/mL	low (ensemble)	very low	low	high	Lacks single-EV resolution; high sample consumption	^77,82–84^
	Luminex	pg/mL	low (ensemble)	moderate	moderate	moderate	high interassay variance; poor reproducibility	^80,81,88^
	MSD (ECL)	high (pg/mL)	low (ensemble)	moderate	moderate	moderate	lacks single-EV resolution	^89,90^
	Western Blot	low (μg/mL)	low (ensemble)	low	low	High	labor-intensive; high sample input	^91,92^
Conventional Omics	LC–MS/MS	variable (ng-μg/mL)	high (proteomic discovery)	excellent	high	low	high cost; complex bioinformatics	^93–95^
	RNA-Seq	high	high (genetic discovery)	excellent	high	low	high cost; demanding computation	^104–106^
Single-entity	Droplet Digital	very high(fM level)	single-entity (Droplet)	high	high	emerging	lower throughput; requires specialized equipment	^113,114^
	Hydrogel	very high(fM level)	single-entity (Particle)	high	moderate	emerging	complex fabrication; limited clinical validation	^115^
	MHPs							
	Nanoarray	very high(fM level)	single-entity (Spatial)	moderate	high	emerging	specialized instrumentation; limited availability	^116^

### 5.1 Bulk Immunoassay

Traditional bulk detection techniques, including ELISA, Luminex, and electrochemiluminescence (ECL), remain the primary tools for BDEV protein quantification due to their established workflows.^77–81^ These platforms are effective for relatively high abundance markers in brain cell subpopulations. For example, ELISA has achieved a detection limit of 0.621 ng/mL for the inflammatory factor C1q in ADEVs.^82^ In clinical cohorts, these tools track α-syn in NDEVs and ODEVs to distinguish PD from Multiple System Atrophy (MSA).^83,84^ Advanced multiplex platforms like Luminex offer even lower limits of detection, reaching 0.7 pg/mL for p181-Tau and 3 pg/mL for Aβ42, allowing researchers to capture pathological signals up to 10 years before clinical diagnosis.^82,85–87^ Furthermore, Luminex enables the simultaneous analysis of panels consisting of 25 inflammatory cytokines in ADEVs, providing a broad molecular profile of neuroinflammation.^88^ In comparison, the ECL technique provides even greater sensitivity and a broader dynamic range than both ELISA and Luminex. Using anti-L1CAM magnetic beads to specifically capture NDEVs, ECL can detect α-syn at much lower concentrations, distinguishing early stage PD patients from healthy controls.^89^

Despite their high sensitivity, bulk methods face significant challenges in analytical resolution and data robustness. Because these assays provide only a population-level average, rare brain-specific signals are often buried under the massive biological noise of systemic circulation. The true limitation is revealed in the high variance of reported concentrations. For instance, while α-syn levels in NDEVs were found to be higher in PD patients than in controls, the fact that the standard deviation often exceeds the mean (107 ± 124 pg/mL in PD versus 58 ± 55 pg/mL in controls) highlights a critical lack of analytical resolution.^83^ This lack of robustness leads to contradictory observations across different studies. While some research shows elevated α-syn in MSA NDEVs (191 ± 131 pg/mL), other groups have reported significantly lower concentrations in MSA compared to PD samples.^83,84,90^ Additionally, many bulk studies are constrained by small sample sizes or the inability to distinguish between pure and mixed disease pathologies.^91^

A major drawback of these bulk platforms is that they sacrifice large volumes of precious clinical samples to reach a detectable signal. Since bulk assays require ensemble-averaged signals to reach a threshold above background noise, they consume significant amounts of biofluid. Conventional protein assays like Western Blotting (WB) have been used to identify glial fibrillary acidic protein (GFAP) in ADEVs for research on Amyotrophic Lateral Sclerosis (ALS), but this method requires significant starting material, such as 1 mL of human serum per sample.^92,93^ In mouse models, researchers often must pool serial blood samples collected over 5 weeks to acquire enough volume for EV isolation and protein quantification.^82^ Clinical studies typically consume 0.25 to 2 mL of plasma or serum per marker to achieve reliable results.^83–87^ This high sample demand quickly depletes biobanks and prevents the true molecular heterogeneity of rare brain-derived subpopulations from being fully captured.

### 5.2 Bulk Proteomics and Genetic Methodologies

To improve detection depth beyond standard assays, Mass Spectrometry (LC–MS/MS) provides the highest multiplexing power for discovering unknown BDEV markers. TMT-based quantitative proteomics has identified over 360 proteins in NDEVs, revealing novel markers like C7 and ZYX linked to Alzheimer’s progression.^94^ In glial research, LC–MS/MS has been vital for identifying proteins related to cell survival and immune responses, such as TREM2 and FTH1, which increase during disease stages.^95–97^ However, bulk proteomics remains sample-hungry and carries a high operational cost. To address this, some studies have replaced expensive magnetic beads with anti-L1CAM antibodies precoated on 96-well plates to achieve high-throughput NDEV enrichment at a lower cost.^98^ The choice of isolation methodology, such as using ExoQuick ULTRA or size exclusion chromatography, also significantly determines the final purity and yield of these markers.^90,94,99^

In nucleic acid detection, specificity and detection speed are the primary metrics for identifying cell-specific RNA. qRT-PCR is the standard tool due to its high sensitivity in measuring miRNAs like miR-146a-5p and miR-223 in BDEVs, which can predict cognitive decline 5 to 7 years in advance.^100–105^ To increase multiplexing, RNA sequencing (RNA-seq) and OpenArrays analyze hundreds of RNA molecules at once from cell-specific samples.^106–108^ For example, sequencing MDEVs has discovered molecules like circZNRF1 that protect neurons in PD models.^106^ Beyond miRNAs, long noncoding RNAs (lncRNAs) like Linc-POU3F3 also serve as biomarkers for PD severity.^109^ While these high-throughput tools offer the best look at the total RNA profile, their high cost and complex data analysis remain major barriers.^110^ Finally, the specificity of these bulk methods relies entirely on the capture strategy. While markers like L1CAM and GLAST are widely used, concerns about L1CAM’s inner-membrane localization or nonspecific expression in other cells suggest that bulk results should be interpreted carefully.^61,111,112^ This ambiguity, particularly the risk of signal overestimation due to soluble protein isoforms, underscores a critical need for validation tools that can transcend ensemble-averaged readouts.

### 5.3 Single-Entity Detection Techniques

While the two-layer evaluation logic establishes a rigorous methodological foundation, the inherent biophysical overlap between EVs and systemic constituents—such as lipoproteins and nonvesicular protein complexes—necessitates verification tools that transcend ensemble-averaged readouts. In this context, the persistent risk of coisolation compromises the specificity required for brain-specific studies, underscoring the need for high-resolution authentication beyond simple recovery metrics. To address these specificity challenges, the analytical paradigm is shifting toward single-entity resolution. Crucially, beyond sensitivity, the primary value of single-entity assays in the evaluation phase lies in their ability to directly visualize marker colocalization on individual particles.^117^ Emerging platforms, including microfluidic-based digital counting and super-resolution imaging, enable the simultaneous detection of multiple antigens—such as a lineage-specific marker and a generic EV tetraspanin—on a single lipid-bilayer nanoparticle.^118,119^ Notably, this spatial correlation effectively bypasses the interference of soluble contaminants, such as the nonvesicular L1CAM isoforms that plague bulk analysis, and ensures that the detected signals represent genuine CNS-derived vesicles. By shifting the evaluation paradigm from analog averaging to event-based verification, these high-resolution tools provide the “biological ground truth” necessary to validate subpopulation purity, establishing a high-fidelity foundation for the diagnostic technologies discussed in this section.

Building upon this logical framework, these approaches digitize EV readouts through physical partitioning or spatial confinement, often coupled with multistage signal amplification. Importantly, practical “single-EV” performance typically relies on operating under a statistically controlled single-occupancy regime and on multimarker colocalization to validate vesicle identity in high-background biofluids. By converting analog molecular signals into countable discrete events, these platforms can mitigate the high and variable background in systemic circulation, enabling the detection of pathogenic signatures otherwise obscured by ensemble averaging.^120^ In practice, current single-EV techniques largely converge on two implementation logics: (i) microfluidic compartmentalization (most commonly droplets) that transforms analog signals into countable events, and (ii) spatially confined signal generation and amplification (e.g., nanoarray/microwell formats). As comprehensively illustrated in Figure 4, these innovative single-entity assays—encompassing droplet microfluidics, rolling circle amplification (RCA), and ultrasensitive on-chip immunoassays (e.g., μTIP-dELISA)—represent a critical leap forward. The overarching comparative advantage of these single-vesicle platforms over conventional methods is their unprecedented ability to eliminate the “averaging effect” of bulk analysis. By digitizing signals at the individual vesicle level, they achieve the ultrahigh sensitivity and spatial resolution required to profile low-abundance target proteins on individual BDEVs, paving the way for high-fidelity clinical diagnostics.

**Figure 4:**
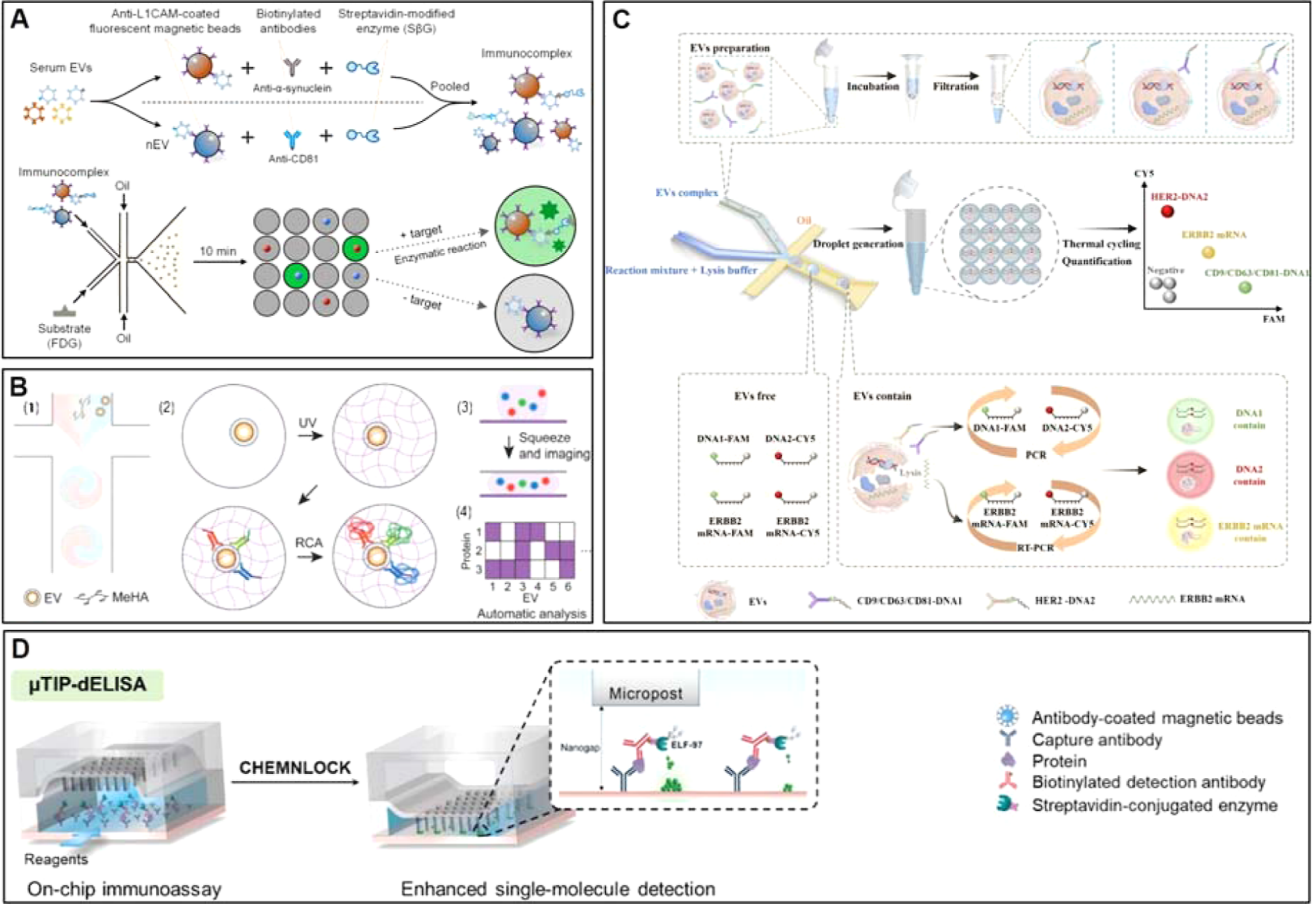
Innovative detection methodologies (A) workflow of the droplet-based microfluidic EVs digital immunoassay. Adapted from ref 113 Available under a CC-BY 4.0 license. Copyright 2025 Elsevier. (B) Overview of the multiplex single EV isolation and analysis with MeHA hydrogel microparticles (MHPs). Adapted from ref 115 Available under a CC-BY 4.0 license. Copyright 2024 John Wiley and Sons. (C) Schematic of combined digital detection of EVs-derived surface protein and nucleic acid at single EV level. Adapted with permission from ref,^114^ Copyright 2025 Elsevier. (D) Schematic workflow of the partition-less digital immunoassay using μTIP-dELISA chip. Adapted with permission from ref 116 Copyright 2025 AMERICAN CHEMICAL SOCIETY.

A representative droplet-based strategy was reported by Yan et al., who designed a microfluidic platform to quantify membrane-associated α-synuclein on serum EVs for discovery and validation of a new marker format for PD diagnostics (Figure 4A).^113^ In this assay, anti-L1CAM–coated fluorescent magnetic beads are used to form complexes with NDEVs, followed by washing and labeling with biotinylated antibodies (200 μL, 0.1 μg/mL) against α-syn and CD81 (a generic EV marker). Streptavidin−β-galactosidase (SβG) is then introduced to generate enzyme-labeled complexes, which are mixed with fluorescein substrate (FDG) and encapsulated into monodisperse droplets (27.9 μm in diameter) using a microfluidic chip. To facilitate robust imaging, droplets are physically compressed to align fluorescence signals within a single focal plane and subsequently imaged by an inverted fluorescence microscope with multichannel detection. In the readout, bead fluorescence in the Cy5 channel (red/blue signal) is used to indicate bead presence and type, while FDG cleavage in the FITC channel generates green fluorescence corresponding to the target-protein signal. This design exemplifies how droplet partitioning, i.e., a nanoconfinement strategy, together with enzyme amplification of its catalyzed fluorescence, can enable sensitive single-EV membrane profiling with limit of detection of 66 CD81^+^ /L1CAM^+^ EVs/mL. With the aid of multiple color-coded immune-magnetic beads (i.e., solid fluorescence), multiplexed profiling can be achieved for simultaneous detection of multiple EV membrane proteins. While Yan et al. showcase superior sensitivity, the reliance on L1CAM challenges biological authenticity. Given L1CAM is known nonvesicular interference, future single-entity assays must prioritize multimarker integration over pure sensitivity to resolve genuine CNS signals from soluble background noise.

Although initially demonstrated using non-neural EV sources, the underlying compartmentalization and multistage amplification principles are readily adaptable for profiling rare BDEV subpopulations under low-input, high-background conditions. Along a conceptually similar route that combines microfluidics with amplification-enabled imaging, the Roh group developed a method using squeezable methacrylated hyaluronic acid hydrogel microparticles (MHPs) for EV profiling (Figure 4B).^115^ MHPs are generated by injecting 2.5% (w/v) methacrylated hyaluronic acid, isolated EVs, and a photoinitiator into a flow-focusing microfluidic droplet generator, followed by photo-cross-linking (1 s UV exposure) in a polymerization chamber to form hydrogel particles. Target proteins are probed by DNA-conjugated antibodies (0.125 μg/mL) that can penetrate the porous mesh of MHPs, triggering RCA to produce long DNA products subsequently visualized by fluorophore-labeled probes. Importantly, MHPs are physically compressed to align RCA products into a single plane for fluorescence microscopy, enabling clear multichannel imaging of signals. Image processing is then performed using a customized software workflow based on CellProfiler to automatically determine biomarker presence/absence and coexpression patterns at the single-EV level. This MHP-based framework highlights the value of combining a permissive porous microenvironment for probe access, nucleic-acid amplification for signal boosting, and computational analysis to extract single-entity phenotypes from high-content images. However, the need for hydrogel fabrication/photo-cross-linking, RCA-based amplification, and customized image-analysis pipelines increases workflow complexity and may pose additional standardization challenges compared with simpler counting-based formats.

Clearly, single-EV platforms are also moving toward multianalyte profiling, which is especially relevant given that many diseases are multifactorial and may not be adequately captured by a single marker. Lin and colleagues demonstrated a droplet-based technique enabling simultaneous detection of EV protein biomarkers and mRNA (Figure 4C).^114^ In their workflow, MCF7-derived EVs are labeled with CD9/CD63/CD81-DNA1 and HER2-DNA2 antibody–DNA conjugates, then coencapsulated with lysis and RT-qPCR reagents into droplets so single-EV lysis drives parallel amplification readouts for membrane proteins (PCR of DNA barcodes, FAM/Cy5) and intravesicular ERBB2 mRNA (RT-qPCR), quantified by multichannel fluorescence imaging and droplet segmentation. This droplet microreactor unifies protein–mRNA measurements at single-EV resolution and can improve subtype discrimination, but its multistep workflow (conjugate labeling/cleanup, droplet stability, thermal cycling, and cluster calling) may limit robustness and translation.

In addition to droplet compartmentalization, microwell/nanoarray strategies provide another route to single-entity digital readouts by confining signal generation to discrete spatial sites. Wen et al. developed a configurable topographic nanoarray platform for Ewing sarcoma (EWS) diagnosis by adapting conventional ELISA into a partition-less digital immunoassay (μTIP-dELISA) (Figure 4D).^116^ EVs derived from EWS cell lines (CHLA-9, CHLA-258) and normal controls (Hs919.T) are pretreated via ultracentrifugation and characterized, followed by lysis with RIPA buffer for 30 min. Lysates diluted in 1% BSA are flowed over a glass surface coated with anti-CD9, anti-NGFR, anti-EZR, and anti-ENO-2 antibodies to immobilize target proteins. Biotinylated detection antibodies, streptavidin-conjugated alkaline phosphatase (SA-ALP), and ELF-97 substrate are then introduced sequentially. By pressing microposts to create surface nanogaps, localized ELF-97 precipitation is generated during a 30 min incubation, producing fluorescent dots that are imaged by confocal microscopy and quantified by dot counting. This nanoarray-enabled dot-counting paradigm effectively translates an analog immunoassay into a digital format and provides a complementary single-EV strategy that does not rely on droplet generation.

Collectively, these examples illustrate a clear trend toward integrated single-EV platforms that combine physical partitioning or spatial confinement, signal amplification (enzymatic turnover, RCA, PCR/qRT-PCR), and multichannel fluorescence imaging with computational analysis.^121,122^ Such single-entity techniques are particularly attractive when disease signals are carried by rare EV subsets or when coexpression patterns and multianalyte signatures are diagnostically informative—conditions under which bulk averaging can be intrinsically limiting.

### 5.4 Bulk vs Single-Entity Detection: Advantages and Trade-offs in Clinical Diagnostics

In clinical diagnostics, bulk EV measurements remain the dominant and most translatable format because they are inherently compatible with routine laboratory infrastructure, standardized operating procedures, and high-throughput cohort testing. Bulk immunoassays and nucleic-acid assays can be readily integrated into established clinical workflows, offering clear advantages in throughput, cost control, and interlaboratory reproducibility. They are therefore well suited for large-scale screening, biomarker validation, and multicenter studies where analytical robustness and operational simplicity are prioritized. However, the core limitation of bulk readouts is that they collapse a heterogeneous EV population into a single ensemble-average value. As a result, clinically informative signals carried by rare EV subpopulations or encoded in combinatorial marker states may be attenuated or even lost, particularly in early stage disease where biomarker abundance is low and biological variation is high.

By contrast, single-entity detection shifts the readout paradigm from analog averaging to event-based quantification, enabling direct interrogation of heterogeneity at the vesicle level. Conceptually, this approach can improve sensitivity by converting low-abundance signals into countable events and can enhance specificity by resolving subpopulation structure and coexpression patterns that are inaccessible to bulk assays. Single-entity methods are thus especially attractive when the diagnostic signal is sparse, when the target fraction represents a minor subset of circulating EVs (e.g., tissue- or brain-derived EVs), or when multimarker phenotypes rather than single biomarkers are expected to provide discrimination. In addition, single-entity platforms can naturally support multiplexing and multianalyte profiling (e.g., protein and nucleic acid information within the same analytical unit), which aligns with the multifactorial nature of many diseases and may improve diagnostic classification and patient stratification.

These benefits, however, come with important trade-offs that currently define the gap between proof-of-concept studies and routine clinical deployment. Single-entity workflows often depend on specialized instrumentation (microfluidics/nanoarrays, high-content imaging), multistep labeling and amplification chemistries, and computational pipelines for segmentation, counting, and quality control. Each of these components can introduce variability and increase the barrier to standardization across operators and sites. In addition, because single-entity assays are highly sensitive to upstream variables, preanalytics—including sample handling, EV isolation/enrichment, nonspecific adsorption control, and matrix suppression—can disproportionately influence performance compared with bulk formats. Operationally, without automation and streamlined sample-to-answer integration, single-entity platforms may face constraints in turnaround time, cost per test, and scalability, all of which are decisive for clinical adoption.

Taken together, bulk and single-entity approaches are best viewed as complementary rather than mutually exclusive. Bulk assays provide a practical backbone for standardized validation and population-level deployment, while single-entity detection offers a higher-resolution layer that can capture heterogeneity, rare-event biology, and combinatorial signatures when bulk averaging becomes limiting. A clinically realistic translation path is therefore a staged strategy: discovery and panel construction can leverage high-content methods, bulk assays can support scalable verification/validation, and single-entity readouts can be deployed to enhance sensitivity and stratification in clinically challenging scenarios. For example, a highly practical clinical workflow could involve an initial high-throughput preliminary screening of large patient cohorts using cost-effective bulk measurements. Subsequently, positive or borderline samples would undergo refined subpopulation analysis and definitive confirmation using high-resolution single-entity assays. Ultimately, the success of this combined approach depends on ensuring that automation, standardization, and robust preanalytics are addressed to meet clinical-grade requirements.

## 6 Conclusion and Discussion

The evidence synthesized in this review underscores the significant potential of BDEV subpopulations as high-fidelity biomarkers for the early diagnosis of NDs. While traditional bulk methodologies remain cornerstones, their reliance on ensemble-averaged signals often obscures rare disease-associated markers. Similarly, genetic tools face challenges related to cost and nonvesicular contamination. Consequently, the field is shifting toward integrated platforms combining high-efficiency separation with single-entity detection.^21,123^ However, the clinical translation of these technologies is hindered by several critical barriers. Technical isolation remains difficult due to the extreme scarcity of BDEVs amidst dominant peripheral backgrounds. Furthermore, the lack of standardized preanalytical protocols and universal reference materials introduces significant variability that complicates validation. Crucially, the field currently lacks a consensus-based, general protocol for definitively authenticating the cellular origins of specific BDEV subpopulations, thereby increasing the risk of signal overlap and compromising the diagnostic specificity of cell-type-specific signatures. To address this specificity bottleneck, developing novel, highly specific biomarkers (e.g., ATP1A3) is paramount to bypass nonvesicular interference from conventional targets like L1CAM. Fundamentally, this biomarker development directly dictates the optimization of next-generation isolation technologies and serves as the fundamental prerequisite for establishing standardized methodologies. Analytically, traditional bulk assays require excessive sample volumes while masking the inherent heterogeneity of individual vesicles. Finally, the deployment of single-entity platforms is constrained by high operational costs and specialized instrumentation, highlighting the need for automated, “sample-to-answer” systems to achieve the scalability required for population-level screening.

Looking forward, the maturation of BDEV-based diagnostics is impeded by the dual challenges of isolation hurdles and biological heterogeneity. The efficient isolation of high-purity BDEV subpopulations remains a technical bottleneck, as these vesicles typically constitute less than 10% of the total circulating EV pool. Future research must prioritize the development of more specific ligands, such as nanobodies or aptamers, and integrate antifouling coating technologies to mitigate the adsorption of nonspecific proteins and peripheral vesicles. By ensuring structural integrity through mild, stimuli-responsive release systems, the field can establish a more reliable foundation for downstream multiomic analysis.^124–126^

The true future of precision neurology lies in the development of an AI-driven multiomic roadmap that transcends the current focus on proteomics and genomics. By integrating data from metabolomics, lipidomics, and imaging markers, multimodal analysis can reveal the comprehensive “molecular fingerprints” necessary for longitudinal disease monitoring. AI and big data analytics will be indispensable in this transition, as AI algorithms can efficiently process complex data sets to identify subtle associations between multidimensional markers that are otherwise inaccessible through traditional statistical methods. Recent pioneering studies have already begun to validate this exact potential. For example, researchers have developed an AI-driven framework utilizing conditional variational autoencoders to autonomously decode multidimensional proteomic signatures from EVs, significantly improving the identification of reliable biomarkers for the early diagnosis of neurodegenerative diseases.^127^ Concurrently in the realm of single-entity analysis, deep learning algorithms have been integrated with high-resolution fluorescence imaging to automatically profile and classify multiple targets on individual vesicles, thereby eliminating the bias of manual feature extraction.^128^ Using AI to optimize the combination of these multiomic features will provide strong support for patient stratification and personalized treatment.^129,130^

Ultimately, the translation of these identified biomarkers into routine clinical practice will culminate in the development of noninvasive POCT technologies.^131^ By simplifying operational workflows and enabling rapid, site-independent testing on noninvasive samples such as blood or saliva, POCT platforms will significantly improve the accessibility and timeliness of ND screening. Achieving this goal requires a staged strategy: leveraging high-resolution single-entity methods for discovery and validation, followed by the deployment of automated, sample-to-answer systems for scalable clinical use. With the continued optimization of sensor technology and AI-driven deconvolution, BDEV evaluation is poised to evolve from a complex research challenge into a transformative diagnostic cornerstone of precision medicine.^130^
